# NF-κB in the crosshairs: Rethinking an old riddle

**DOI:** 10.1016/j.biocel.2017.12.020

**Published:** 2018-02

**Authors:** Jason Bennett, Daria Capece, Federica Begalli, Daniela Verzella, Daniel D’Andrea, Laura Tornatore, Guido Franzoso

**Affiliations:** Centre for Cell Signalling and Inflammation, Department of Medicine, Imperial College London, London W12 0NN, UK

**Keywords:** BCL-2, B-cell lymphoma 2, BCL-X_L_, B-cell lymphoma-extra large, BFL-1, Bcl-2-related gene expressed in foetal liver, cIAP, cellular inhibitor of apoptosis, cFLIP, cellular FLICE-inhibitory protein, CLL, chronic lymphocytic leukaemia, DIABLO, direct inhibitor of apoptosis protein (IAP)-binding protein with low pI, DLBCL, diffuse large B-cell lymphoma, FDA, US Food and Drug Administration, FLICE, FADD-like IL-1β-converting enzyme, GADD45β, growth arrest and DNA damage inducible β, GBM, glioblastoma multiforme, IKK, IκBα kinase, IL-1β, interleukin-1β, IκBα, nuclear factor of κ light polypeptide gene enhancer in B-cells inhibitor α, JNK, c-Jun N-terminal kinase, MALT, mucosa-associated lymphoid tissue, MAP, mitogen activated protein, MCL1, myeloid cell leukaemia sequence 1, NF-κB, nuclear factor κ B (nuclear factor binding to the κ-light-chain-enhancer B site), PTEN, phosphatase and tensin homolog, RAS, rat sarcoma virus oncogene, SCF, SKP1-Cullin 1-F-box protein, SMAC, second mitochondria-derived activator of caspases, TME, tumour microenvironment, βTrCPP, β-transducin repeat-containing proteint, XIAP, X chromosome-linked inhibitor of apoptosis protein, Cancer, NF-κB, B, IKKβ, GADD45β, DTP3

## Abstract

Constitutive NF-κB signalling has been implicated in the pathogenesis of most human malignancies and virtually all non-malignant pathologies. Accordingly, the NF-κB pathway has been aggressively pursued as an attractive therapeutic target for drug discovery. However, the severe on-target toxicities associated with systemic NF-κB inhibition have thus far precluded the development of a clinically useful, NF-κB-targeting medicine as a way to treat patients with either oncological or non-oncological diseases. This minireview discusses some of the more promising approaches currently being developed to circumvent the preclusive safety liabilities of global NF-κB blockade by selectively targeting pathogenic NF-κB signalling in cancer, while preserving the multiple physiological functions of NF-κB in host defence responses and tissue homeostasis.

## Highlights

• NF-κB transcription factors are central coordinating regulators of immunity, inflammation and cell survival.
• NF-κB pathway is aberrantly and stably activated in cancer.
• The ubiquitous presence and pleiotropic physiological role of NF-κB dimers have thus far prevented the development of any clinically useful NF-κB inhibitor.
• Emerging therapeutic approaches aim to achieve the cancer-selective inhibition of the NF-κB pathway as a way to overcome the preclusive toxicities of conventional IKKβ/NF-κB-targeting drugs.

## 1 Introduction

The anticancer arsenal has traditionally consisted of a limited number of broadly active cytotoxic chemotherapeutics characterised by a small therapeutic index and a minimal capacity to discriminate between malignant and normal cells. Over the past 25 years, fundamental advances in the field of molecular oncology and the understanding of many of the core mechanisms driving oncogenesis have enabled the generation of rationally designed, targeted therapies which selectively interfere with discrete oncogenic effectors, thereby opening the door to an era of stratified oncology and, consequently, revolutionising the clinical management of cancer patients. Indeed, the oncology field is currently undergoing a new revolution with the boom of anticancer immunotherapies capable of producing long-term remissions and even curative outcomes, breaking away from traditional paradigms by targeting the non-malignant, rather than malignant, cell components within tumours (Hodi et al., 2010; Brahmer et al., 2012).

The scientific breakthroughs of the past few decades have enabled the creation of a new generation of anticancer medicines, which couple greater specificity with reduced adverse effects, thus equipping the current anticancer armoury with multiple classes of new agents which selectively interfere with a wide spectrum of discrete drivers of oncogenesis. However, while an ever-growing number of cancer-driving mechanisms and signalling pathways have thus far been successfully pharmacologically targeted, leading to improved clinical outcomes in oncology, a select group of other pathways have proven defiant to therapeutic intervention. Among these, the NF-κB pathway stands out as perhaps the most illustrious example and arguably the one that has coalesced the greatest frustration and disappointment.

Ubiquitous NF-κB transcription factors are central coordinating regulators of the host defence responses to stress, injury and infection (Hayden and Ghosh, 2012; Zhang et al., 2017). In addition to fulfilling these elemental physiological roles, NF-κB contributes to the pathogenesis of most of the chief threats to global human health, including cancer, atherosclerosis, diabetes and chronic inflammatory diseases (Xia et al., 2014; DiDonato et al., 2012). Aberrant NF-κB signalling is a hallmark of the large majority of human cancers, where it drives oncogenesis, disease recurrence and therapy resistance, largely by regulating genes that suppress malignant cell apoptosis and govern inflammation in the tumour microenvironment (TME) (Xia et al., 2014; DiDonato et al., 2012). Unsurprisingly, owing to these pivotal pathogenic roles of NF-κB, the targeting of the NF-κB pathway has been a paramount objective of the pharmaceutical industry and the focus of worldwide research efforts for the past 25 years, as a means to improve the clinical management of both oncological and non-oncological patients, especially within particularly refractory disease indications (Gilmore and Herscovitch, 2006; Begalli et al., 2017). However, as best illustrated by the ill-fated pursuit of a clinically useful inhibitor of IκBα kinase (IKK)β, the kinase responsible for phosphorylating IκB proteins and enabling nuclear NF-κB translocation (Hayden and Ghosh, 2012), achieving this goal has to this day proven an insurmountable problem, owing to the failure of traditional IKK/NF-κB-targeting strategies to preserve the pleiotropic and ubiquitous physiologic functions of NF-κB (Greten et al., 2007; Hsu et al., 2011). This minireview offers a glimpse into some of the more promising emerging approaches currently being considered to circumvent these inherent limitations of conventional NF-κB inhibitors, with a focus on oncology.

### 1.1 The futile pursuit of a specific NF-κB inhibitor: an historical perspective on an obstinate conundrum

**Fig. 1:**
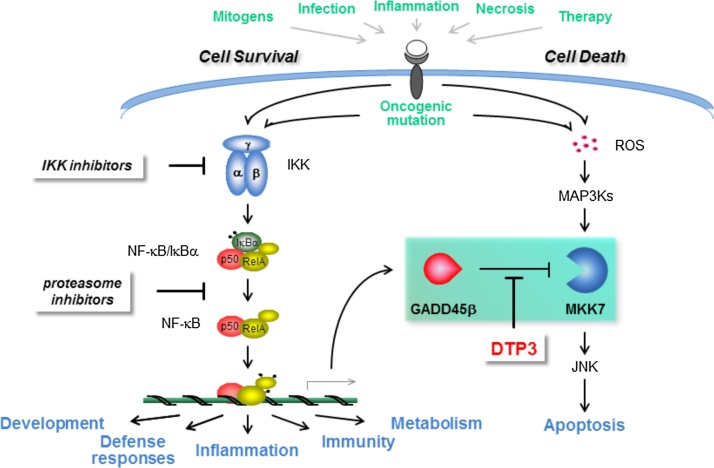
The cancer-selective strategy to target the NF-κB signalling pathway. Schematic representation of the canonical pathway of NF-κB activation. Depicted in black are the main conventional therapeutic strategies, which have thus far been used to generate pharmacological NF-κB inhibitors. Also depicted in red is one of the emerging approaches aimed at developing a therapeutic inhibitor of a functionally critical and cancer cell-restricted downstream effector of the pathogenic survival axis of the NF-κB pathway. Also shown is the D-tripeptide inhibitor of the GADD45β/MKK7 complex, DTP3, which selectively targets this GADD45β-dependent survival axis of the NF-κB pathway, yielding cancer cell-selective therapeutic activity, thereby circumventing the preclusive limitations of global IKKβ/NF-κB inhibitors.

Following its discovery by Baltimore and colleagues in 1986, as a nuclear factor binding to a conserved DNA enhancer region of the κ light-chain immunoglobulin gene in activated B cells, the NF-κB signalling pathway soon became the paradigm of the rapid response mechanisms governing the cellular adaptation to environmental or internal changes by regulating the expression of versatile, inducible genetic programmes (Hayden and Ghosh, 2012; Zhang et al., 2017). In mammals, NF-κB comprises a family of five proteins, known as RelA/p65, RelB, c-Rel, p50/NF-κB1 (p105), and p52/NF-κB2 (p100), which can form multiple combinations of distinct heterodimeric and homodimeric complexes, the most abundant of which is the RelA/p50 heterodimer (Fig. 1) (Begalli et al., 2017; Zhang et al., 2017). In cells, these complexes are normally held in a state of latency in the cytosol, where they are bound to IκB-family inhibitory proteins and can be activated in response to inflammatory stimuli, microbial products and a broad spectrum of other signals, which cause the site-specific phosphorylation of IκBs by the IKK complex, leading to the sequential polyubiquitination and proteolysis of phosphorylated IκBs by the SCF^βTrCP^ E3 ubiquitin-protein ligase complex and the 26S proteasome, respectively (Hayden and Ghosh, 2012; Begalli et al., 2017; Winston et al., 1999; Spencer et al., 1999). Thereafter, the liberated NF-κB dimers enter the nucleus where they bind to distinct DNA elements, known as κB sites, to coordinate the expression of a diverse array of inflammatory mediators, immunoregulators, apoptosis inhibitors, developmental signals and numerous other factors orchestrating the immune and inflammatory responses through a process that is normally transient and self-limiting (Hayden and Ghosh, 2012; Zhang et al., 2017). Over the past three decades, ubiquitous NF-κB dimers have been shown to activate and repress hundreds of different target genes mediating these functions, bestowing upon this family of transcription factors a remarkable capability to inducibly alter cell physiology (Hayden and Ghosh, 2012; Zhang et al., 2017). Interestingly, notwithstanding its ubiquitous nature, the NF-κB pathway has also been ascribed a sophisticated capacity to achieve a remarkably wide degree of contextual diversity in the transcriptional programmes it activates in any given cell, upon nuclear translocation, dependent upon the tissue type and specific biological circumstances in which it is induced (Zhang et al., 2017).

Given the multitude of stimuli that can activate NF-κB and the broad spectrum of functions that NF-κB plays in different tissues, it is unsurprising that several feedback mechanisms have evolved to ensure the tight control and timely termination of physiological NF-κB signalling as a way to enable the prompt return to homeostasis and prevent excessive inflammation, tissue damage and the development of malignancy (Hayden and Ghosh, 2012; Zhang et al., 2017; Begalli et al., 2017). Indeed, excessive and stable IKK/NF-κB activation is a typifying feature of a wide range of pathological states, including cancer. Whereas in certain malignancies, such as multiple myeloma, diffuse large B-cell lymphoma (DLBCL), mucosa associated lymphoid tissue (MALT) lymphoma and glioblastoma multiforme (GBM), NF-κB is often constitutively activated by recurrent genetic alterations targeting upstream components of the NF-κB pathway, in the large majority of solid tumours and certain haematological malignancies, such alterations of the NF-κB pathway are relatively infrequent (DiDonato et al., 2012; Annunziata et al., 2007; Keats et al., 2007; Pasqualucci et al., 2001; Bredel et al., 2011). Accordingly, in these cancers, aberrant NF-κB activation generally stems from genetic abnormalities targeting conventional tumour-suppressor and oncogenic mechanisms, such as *RAS* and *PTEN* mutations, and/or the steady exposure of tumour cells to inflammatory stimuli and other cues emanating from the TME (DiDonato et al., 2012). These findings, and an accompanying extensive body of other genetic, biochemical and clinical evidence, provide a compelling rationale for therapeutically blocking constitutive NF-κB signalling in a wide range of human cancers in areas of current unmet need. Moreover, there is a strong rationale for developing NF-κB-targeting therapeutics to treat numerous non-malignant human pathologies, such as diabetes, autoimmune disorders, and chronic inflammatory diseases, owing to the central role of NF-κB signalling in governing inflammation, and the underlying low-grade inflammatory reaction that propagates the pathogenesis of these and virtually all other human illnesses (Xia et al., 2014; DiDonato et al., 2012). Notwithstanding, to this day – more than 30 years since the discovery of NF-κB and despite an aggressive and persevering effort by the pharmaceutical industry over the past 25 years – no specific NF-κB inhibitor has been clinically approved, due to the preclusive on-target toxicities associated with the systemic inhibition of NF-κB (Gilmore and Herscovitch, 2006; Begalli et al., 2017).

Owing to its central role as the downstream signal-integration hub for the pathways of NF-κB activation, IKKβ bore the brunt of the drug discovery effort to inhibit pathological NF-κB signalling since its discovery in 1996 (Fig. 1). Nonetheless, while the initial impetus did succeed in generating a large array of specific molecules and multiple candidate therapeutics, this effort eventually came to an inevitable abrupt end, as soon as IKKβ inhibitors were evaluated in animal models and early-phase clinical trials (DiDonato et al., 2012; Greten et al., 2007; Hsu et al., 2011). In a seminal paper published in 2007, Karin and colleagues demonstrated that the pharmacological inhibition of IKKβ increases IL-1β secretion by myeloid cells, owing to an enhanced processing of pro-IL-1β by caspase 1, leading to overt systemic inflammation and increased animal lethality (Greten et al., 2007). In addition to this unanticipated, dose-limiting adverse effect, subsequently confirmed in human studies, global IKKβ/NF-κB inhibition produced a series of other adverse effects, including immunodeficiencies, hepatotoxicity and a potentially increased risk of malignancies arising from tissues such as the liver and the skin, reflecting the essential roles of NF-κB in innate and adaptive immune responses and tissue homeostasis (DiDonato et al., 2012; Greten et al., 2007; Hsu et al., 2011). Eventually, after the initial, short-lived enthusiasm, these findings irrevocably halted any further significant clinical development of IKKβ/NF-κB inhibitors, as demonstrated by the recent dramatic decline in new patent applications relating to these agents (Begalli et al., 2017).

Another class of drugs originally developed to therapeutically target pathological IKKβ/NF-κB signalling are proteasome inhibitors, which stabilise IκB proteins, thereby preventing nuclear NF-κB translocation by interfering with the proteolytic activity of the proteasome (Fig. 1) (Zhang et al., 2017; Manasanch and Orlowski, 2017). These molecules, as well as immunomodulatory drugs (IMiDs), are known to impact upon NF-κB signalling and have found broad clinical indication in multiple myeloma and a handful of other malignant pathologies. However, both classes of drugs display broad biological activities, lack any specificity for NF-κB, and, importantly, afford clinical benefit in these indications via a mechanism unrelated to the NF-κB pathway (Manasanch and Orlowski, 2017; Richardson, 2010). Consequently, there remains an urgent need for a fresh and entirely different approach to safely targeting the NF-κB pathway in human diseases.

### 1.2 Embracing complexity as a path to achieve the safe therapeutic inhibition of the NF-κB pathway

Historically, the insurmountable problem with conventional NF-κB-targeting strategies has been to achieve the contextual, tissue-specific inhibition of the NF-κB pathogenic activity, while preserving the pleiotropic and ubiquitous physiological functions of NF-κB, including its functions in immunity and inflammation (DiDonato et al., 2012). Since the best documented activity of NF-κB in oncogenesis is to upregulate genes that suppress cancer-cell apoptosis, and despite its ubiquitous nature, NF-κB signalling elicits transcriptional programmes that vary considerably depending upon the type of tissue and activating stimulus, we sought to target a non-redundant, cancer cell-specific downstream effector of this oncogenic NF-κB-mediated survival function, rather than NF-κB itself (Fig. 1) (Begalli et al., 2017; Annunziata et al., 2007; Keats et al., 2007; Bennett et al., 2013). We postulated that this strategy could provide a comparably effective, yet considerably safer alternative to conventional IKKβ/NF-κB-targeting drugs, thus circumventing the dose-limiting toxicities of systemic IKKβ/NF-κB inhibition.

Our group recently tested this hypothesis in the context of multiple myeloma, a malignancy of plasma cells (PC) responsible for almost 2% of all cancer deaths and representing the paradigm of NF-κB-driven cancers (Annunziata et al., 2007; Keats et al., 2007). Despite the recent introduction of new treatments, almost all multiple myeloma patients eventually relapse and/or develop drug resistance. Consequently, the management of these patients remains a significant medical problem. Given its paramount importance in disease pathogenesis, the NF-κB pathway provides an attractive therapeutic target in multiple myeloma. Indeed, virtually all clinical cases of this neoplasia display constitutive NF-κB signalling and elevated NF-κB target-gene signature, leading to malignant cell addiction to nuclear NF-κB activity for survival and sensitivity to apoptosis upon IKKβ/NF-κB inhibition (Annunziata et al., 2007; Keats et al., 2007).

Our group, as well as others, previously reported that NF-κB inhibits apoptosis, at least in part, by suppressing the exaggerated activation of the JNK MAPK pathway through a mechanism that involves the transcriptional upregulation of *Growth Arrest and DNA Damage 45B* (*GADD45B*), a member of the GADD45 family of inducible genes, and other downstream effectors, such as *X chromosome-linked inhibitor of apoptosis protein* (*XIAP*) (Jin et al., 2002; De Smaele et al., 2001; Lin and Karin, 2003). Subsequent studies demonstrated that prolonged JNK activation leads to apoptosis, in part, by causing the phosphorylation-dependent activation of the E3 ubiquitin ligase, Itch, which in turn promotes the polyubiquitination and subsequent proteasome-mediated degradation of the caspase 8/10 inhibitor, cellular FLICE (FADD-like IL-1β-converting enzyme)-inhibitory protein (c-FLIP), leading to caspase 8/10 activation and ultimately cell death (Bennett et al., 2013). Prolonged JNK activation has been shown to also enhance the activity of several proapoptotic members of the B-cell lymphoma (BCL)-2 family of proteins, such as Bim and Bmf, by promoting their release from sequestered cytoplasmic pools normally bound to dynein and myosin V motor complexes (Kuwana and Newmeyer, 2003).

Recently, we identified the complex formed by GADD45β and the JNK kinase, MKK7, as an essential survival module dependent on constitutive NF-κB signalling and a novel therapeutic target in multiple myeloma (Fig. 1) (De Smaele et al., 2001; Papa et al., 2004; Tornatore et al., 2014; Tornatore et al., 2015; Papa et al., 2007). We demonstrated that *GADD45B* is upregulated in multiple myeloma cells by constitutive NF-κB activation, promotes malignant cell survival by suppressing proapoptotic MKK7/JNK signalling through its direct binding to and inhibition of MKK7, and is associated with poor clinical outcome in multiple myeloma patients (Tornatore et al., 2014; Tornatore et al., 2015). Importantly, most healthy cells do not constitutively express GADD45β, nor rely on GADD45β for their survival, and, unlike mice lacking IKKβ, any other IKK component, or the NF-κB subunit, RelA – which all die during late embryogenesis – Gadd45β-deficient mice are viable, fertile, and die of old age (Lu et al., 2004). Accordingly, we hypothesised that, in contrast to systemic NF-κB blockade, pharmacological GADD45β inhibition would be well tolerated, *in vivo*. Therefore, we sought to selectively target the NF-κB oncogenic function in multiple myeloma cells by inhibiting the GADD45β/MKK7 survival module downstream in the NF-κB pathway.

By screening a simplified combinatorial tetrapeptide library, followed by chemical optimisation, we developed the pharmacological D-tripeptide inhibitor, DTP3, which specifically binds to MKK7 with high affinity, disrupting the GADD45β/MKK7 interaction, and, as a result, selectively kills multiple myeloma cells by inducing MKK7/JNK-dependent apoptosis (Fig. 1) (Tornatore et al., 2014; Tornatore et al., 2015). We showed that, due to its target cell-specific mode of action, DTP3 displays potent and cancer-selective therapeutic activity against multiple myeloma cell lines and malignant PCs from multiple myeloma patients and, importantly, is not toxic to normal cells. Owing to these properties, DTP3 exhibited a more than 100-fold higher cancer-cell specificity than either proteasome or IKKβ inhibitors in primary human cells, *ex vivo*. Notably, as a result of this cancer cell-selective specificity, DTP3 caused a complete regression of established tumour xenografts, extending host survival in mouse models of multiple myeloma, upon intravenous administration, with excellent tolerability and no adverse effects at the therapeutic dose levels (Tornatore et al., 2014; Tornatore et al., 2015).

Further toxicology studies demonstrated that DTP3 was well tolerated in both rodent and non-rodent species, upon daily repeated-dose administration at high doses for 28 days, exhibiting no target organs of toxicity and no significant adverse effects, resulting in a wide therapeutic index and exposing no risk for its clinical progression. Accordingly, we are currently conducting the first-in-human phase-I/IIa study of DTP3 in patients with refractory or relapsed multiple myeloma. Upon an initial evaluation, DTP3 demonstrated clinical safety and tolerability at all dose levels investigated thus far, alongside a cancer-selective pharmacodynamic response, in highly refractory oncological patients and as a single agent. Future, larger clinical studies will determine the long-term clinical safety and therapeutic efficacy of DTP3 in patients with multiple myeloma and potentially other types of cancer in which DTP3 is indicated. While DTP3 has thus far produced no significant adverse effects in preclinical models or multiple myeloma patients, and unlike sensitive tumour cells, most normal cells do not constitutively express GADD45β, nor display spontaneous MKK7/JNK activation upon GADD45β inhibition, there remains a possibility that DTP3 administration will result in an exacerbation of MKK7/JNK signalling at sites of pre-existing inflammation, thus aggravating chronic inflammatory comorbidities and/or increasing the risk of autoimmune diseases. Further clinical studies will also consolidate the companion stratification strategy to select those patient subsets who will optimally respond to DTP3 and determine whether and, eventually, how rapidly responding tumours develop resistance to DTP3, for instance by acquiring *MKK7* gene mutations or functionally redundant, GADD45β-independent antiapoptotic mechanisms. Notwithstanding, together with the compelling preclinical package, these highly encouraging initial clinical results introduce an unprecedented therapeutic mode of action – possessing none of the preclusive safety constraints of conventional IKKβ/NF-κB inhibitors – into clinical oncology and bode well for the ultimate clinical success of DTP3 as a safe and highly effective NF-κB-targeting therapeutic. These results also provide initial proof-of-concept for a safe and cancer-selective NF-κB-targeting strategy as a novel anticancer therapy which promises to be of profound benefit for patients with multiple myeloma and, potentially, other cancers where NF-κB drives oncogenesis via GADD45β (Fig. 1) (Karin, 2014).

Importantly, the same principle we developed of therapeutically inhibiting a cancer-restricted axis of the NF-κB pathway, rather than NF-κB globally, could be also applied to selectively targeting the NF-κB oncogenic function in GADD45β-independent malignancies and, plausibly, in the context of non-malignant NF-κB-driven diseases (Tornatore et al., 2014). Indeed, the NF-κB survival function is mediated by the upregulation of a diverse group of antiapoptotic target genes, which are independently transcriptionally regulated in a tissue- and stimulus-specific manner. Therefore, this NF-κB function is neither exclusively dependent upon GADD45β induction, nor is it necessarily dependent upon the suppression of JNK signalling (Xia et al., 2014; Bennett et al., 2013). For example, NF-κB has been shown to transcriptionally regulate the coding genes for several antiapoptotic members of the BCL-2 family, including B-cell lymphoma-extra large (Bcl-X_L_), myeloid cell leukaemia sequence 1 (MCL1), B-cell lymphoma 2-related protein A1 (BCL2-A1)/Bcl-2-related gene expressed in foetal liver (BFL-1), and, in certain biological contexts, BCL-2 itself. These proteins are involved in maintaining the outer mitochondrial membrane integrity, thereby preventing the release of cytochrome c and other proapoptotic mitochondrial factors, such as second mitochondria-derived activator of caspases (SMAC)/direct inhibitor of apoptosis protein (IAP)-binding protein with Low pI (DIABLO), into the cytosol, ultimately inhibiting the onset of cell death (Vogler, 2014). These antiapoptotic members of the BCL-2 family are also known to promote cancer-cell survival in various types of haematological and solid malignancy (Catz and Johnson, 2001; Cang et al., 2015). Notably, BCL-2-targeting drugs have been successfully developed outside the scope of blocking oncogenic NF-κB signalling, and drugs in this class, including the first-in-class BCL-2-family inhibitor, ABT-199 (venetoclax), have been granted breakthrough status designation by the FDA for treating subsets of patients with relapsed or refractory chronic lymphoid leukaemia (CLL) (Cang et al., 2015; Levy and Claxton, 2017). Therefore, these agents could be additionally developed to therapeutically target pathogenic NF-κB signalling in oncological situations in which NF-κB promotes malignant cell survival through the upregulation of BCL-2-like factors.

A potential alternative strategy to selectively target the NF-κB pathway in human cancer involves the inhibition of upstream signalling mechanisms that drive oncogenesis by virtue of their role in regulating NF-κB activation. For instance, the members of the IAP-family of E3 ubiquitin ligases, c-IAP1 and c-IAP2, have been shown to contribute to NF-κB activation by tumour necrosis factor (TNF)α and other stimuli through its binding to TNF receptor-associated factor (TRAF)-family proteins through their baculovirus IAP repeat (BIR) domain, leading to the polyubiquitination of their signalling substrates, including TRAF proteins themselves, to enable the ubiquitin-mediated assembly of multimeric, receptor-specific protein scaffolds, in which the IKK complex is brought into physical proximity of the transforming growth factor β-activated kinase (TAK)1 kinase complex, thereby resulting in IKKβ activation by TAK1-mediated phosphorylation. As well as XIAP, which is also involved in the recruitment of the TAK1 complex to the NF-κB signalosome, c-IAP1/2 proteins have been found to be highly expressed in a subset of human cancers, where they can promote NF-κB activation and cancer therapy resistance. Therefore, since small-molecule dual antagonists of c-IAP1 and XIAP, such as ASTX660, have been recently progressed into phase-II clinical studies in patients with various types of advanced haematological or solid cancer, including DLBCL, T-cell lymphoma, head and neck squamous cell carcinoma (HNSCC) and cervical carcinoma, these therapeutic molecules could be further developed to selectively target oncogenic NF-κB signalling in those clinical cases in which NF-κB is activated by the overexpression of c-IAP1 and/or XIAP proteins. This handful of examples underscore how recent advances in the understanding of the biological functions and regulation of the NF-κB pathway, and the contextual make-up of the genetic programmes NF-κB selectively elicits in cancer cells, are currently providing tangible new opportunities for targeted therapeutic interventions in different areas of unmet need across the oncological landscape.

## 2 Conclusions

Owing to its central role in disease pathogenesis, the NF-κB pathway has been pursued for decades as an attractive target for therapeutic intervention. Yet, despite the clear need for a specific NF-κB inhibitor to treat a broad range of human diseases, developing such a molecule has so far presented an impenetrable riddle, due to the need to confront a ubiquitous signalling pathway that has many elemental physiological functions. This has resulted in the dismaying absence of an NF-κB-targeting drug from the current pharmacopoeia. Despite this bleak reality, the past three decades have seen a succession of fundamental advances in the understanding of the intertwined signalling networks governing NF-κB activation, the myriad of cellular functions the NF-κB pathway embodies, and the diverse transcriptional programmes it contextually governs in any given cell, whether in normal or unhealthy tissues. Indeed, these advances are now providing important clues to finally untangle the NF-κB conundrum, and these clues, in turn, are beginning to translate into targeted and much safer alternatives to global NF-κB blockade as a way to effectively treat patients, both within and outside oncology. While the initial successes in the experimental settings have yet to transform into a clear healthcare benefit, the conceptual revolution conveyed in the approach to therapeutically targeting the NF-κB pathway is already providing tangible opportunities for developing effective new treatments in refractory disease indications. Indeed, if there is a lesson to be learnt from these initial successes, it is that the deep-rooted complexity in the NF-κB pathway may well hold the key to unlocking the gateway to generating clinically useful NF-κB-targeting medicines. Therefore, embracing, rather than evading, this complexity appears to the path to follow in order to finally seize the therapeutic potential still captured in the NF-κB pathway.

Recent reports suggest that one way of achieving this goal would be to exploit the contextual diversity of the transcriptional programmes NF-κB elicits in different cell types and, accordingly, inhibit the non-redundant, tissue-restricted downstream effectors of the NF-κB pathogenic functions. Although further clinical evaluation will ultimately determine its safety and clinical benefit, this approach has already added a firm string to the bow of the promising new therapies being developed to selectively inhibit NF-κB signalling in cancer. Additional attractive strategies are also appearing on the horizon of realising the contextual, cancer-cell selective inhibition of the NF-κB pathway by exploiting, for instance, the tissue-specific signalling mechanisms governing the contextual NF-κB activation in cells. Future research will tell whether NF-κB inhibitors will ever become part of the available anticancer arsenal. However, the significant advances recently made in this direction bode well for enabling this new reality in the near future.

## Conflict of interest

The authors declare no conflict of interest.
